# Prognostic value of the delta neutrophil index in pediatric cardiac arrest

**DOI:** 10.1038/s41598-020-60126-y

**Published:** 2020-02-26

**Authors:** Seo Hee Yoon, Eun Ju Lee, Jinae Lee, Moon Kyu Kim, Jong Gyun Ahn

**Affiliations:** 1 Department of Pediatrics, Severance Children’s Hospital, Yonsei University College of Medicine, Seoul, Korea; 2 Department of Biomedical Systems Informatics, Biostatistics Collaboration Unit, Yonsei University College of Medicine, Seoul, Korea

**Keywords:** Predictive markers, Prognostic markers, Paediatric research

## Abstract

The delta neutrophil index (DNI), which reflects the ratio of circulating immature neutrophils, has been reported to be highly predictive of mortality in systemic inflammation. We investigated the prognostic significance of DNI value for early mortality and neurologic outcomes after pediatric cardiac arrest (CA). We retrospectively analyzed the data of eligible patients (<19 years in age). Among 85 patients, 55 subjects (64.7%) survived and 36 (42.4%) showed good outcomes at 30 days after CA. Cox regression analysis revealed that the DNI values immediately after the return of spontaneous circulation, at 24 hours and 48 hours after CA, were related to an increased risk for death within 30 days after CA (*P* < 0.001). A DNI value of higher than 3.3% at 24 hours could significantly predict both 30-day mortality (hazard ratio: 11.8; *P* < 0.001) and neurologic outcomes (odds ratio: 8.04; *P* = 0.003). The C statistic for multivariable prediction models for 30-day mortality (incorporating DNI at 24 hours, compression time, and serum sodium level) was 0.799, and the area under the receiver operating characteristic curve of DNI at 24 hours for poor neurologic outcome was 0.871. Higher DNI was independently associated with 30-day mortality and poor neurologic outcomes after pediatric CA.

## Introduction

Cardiac arrest (CA) is a life-threatening condition that results in whole-body ischemia-reperfusion syndrome^1,2^. Despite recent advances in treatment strategies, mortality rates after CA in children still range from 35% to 62%;^2–6^ furthermore, about 25% to 84% of children survive to discharge but with poor functional outcomes^6–9^. Since children have greater life expectancy compared to adults, they also have the potential to live long with sequelae after ischemic injury^10^.

Early and precise outcome prediction after CA is mandatory for close monitoring, proper treatment planning, and family support^2^. Pupillary reflex, electroencephalographic data and several brain specific biomarkers have previously been suggested to be capable of predicting neurologic prognosis after CA in both adults and children^11–14^. However, physical examination could be imprecise due to sedatives or paralytics administered after CA^11,15^, and no single parameter has yet been identified as having advantages over others^2,11^. The combination of multiple biomarkers with clinical parameters was reported to be highly predictive of survival and neurological outcomes in adult out-of-hospital cardiac arrest (OHCA)^16^, but biomarkers usually require specialized laboratory equipment for assay and therefore cannot be routinely measured in a typical hospital setting^11,14,16^.

The delta neutrophil index (DNI) reflects the proportion of circulating immature neutrophils. DNI can easily and quickly be obtained from the complete blood count (CBC) by a specific automated blood cell analyzer^17,18^. Several studies have demonstrated that DNI could predict severity in various systemic inflammation statuses in adults such as sepsis, vasculitis, upper gastrointestinal bleeding, ST-elevation myocardial infarction, and OHCA^17–22^. However, studies assessing the prognostic value of DNI in pediatric CA still remain limited. The aim of this study was to assess the clinical usefulness of DNI as a prognostic marker of short-term mortality and neurologic outcomes after CA in pediatric patients.

## Results

### Patient enrolment and characteristics

**Figure 1:**
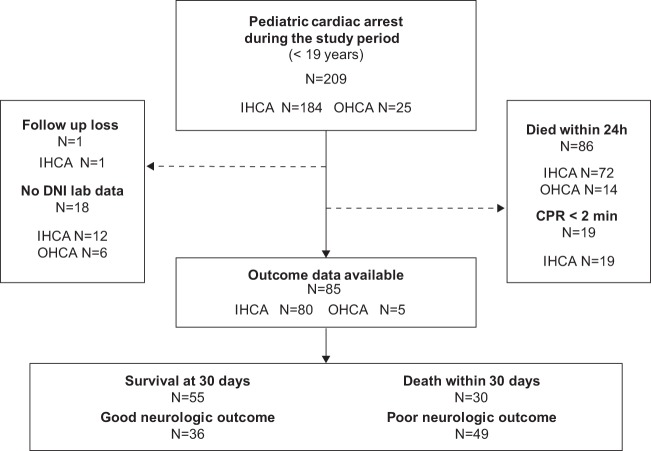
Flowchart of patient inclusion and exclusion. DNI, delta neutrophil index; CPR, cardiopulmonary resuscitation; IHCA, in-hospital cardiac arrest; OHCA, out-of-hospital cardiac arrest.

Of the 209 patients with CA identified during the study period, a total of 123 (58.9%) patients survived at least 24 hours after ROSC (return of spontaneous circulation), while 86 died in the first 24 hours. We excluded 38 patients due to receiving cardiopulmonary resuscitation (CPR) for less than 2 minutes (n = 19), having no available DNI value (n = 18), and loss to follow-up (n = 1) (Fig. 1). The remaining 85 eligible patients were divided according to their mortality and neurologic outcomes at 30 days after CA. Fifty-five of the 85 subjects (64.7%) survived, and 36 (42.4%) had good neurologic outcomes (PCPC; Pediatric Cerebral Performance Category score = 1–3) at 30 days after CA (Table 1). The baseline clinical characteristics stratified by outcomes are shown in Table 1.

**Table 1:** Baseline clinical characteristics stratified by outcomes.

Variables	Total(N = 85) [n]	30-day mortality		*P*-value	30-day PCPC score		*P*-value
		Survival (N = 55) [n]	Death (N = 30) [n]		Good (N = 36) [n]	Poor (N = 49) [n]	
Time of arrest				0.757			0.649
Day	64(75.29)	42(76.36)	22(73.33)		28(77.78)	36(73.47)	
Night	21(24.71)	13(23.64)	8(26.67)		8(22.22)	13(26.53)	
Female sex (n,%)	30(35.29)	18(32.73)	12(40)	0.503	12(33.33)	18(36.73)	0.746
Age, years	2(0.8~9.5)[85]	2.5(1.1~7.8)[55]	2(0.2~11.5)[30]	0.982	3.5(1.1~10)[36]	2(0.6~9)[49]	0.637
IHCA/OHCA				0.340			>0.999
In hospital	80(94.12)	53(96.36)	27(90)		34(94.44)	46(93.88)	
Out of hospital	5(5.88)	2(3.64)	3(10)		2(5.56)	3(6.12)	
Initial rhythm (n,%)				0.409			0.332
Asystole	23(27.06)	12(21.82)	11(36.67)		9(25)	14(28.57)	
PEA	20(23.53)	15(27.27)	5(16.67)		11(30.56)	9(18.37)	
VT/VF	6(7.06)	5(9.09)	1(3.33)		4(11.11)	2(4.08)	
Bradycardia	34(40)	21(38.18)	13(43.33)		11(30.56)	23(46.94)	
Unknown	2(2.35)	2(3.64)	0(0)		1(2.78)	1(2.04)	
CPR duration, min	9(4~24)[85]	6(3~14)[55]	18.5(7~40)[30]	0.002^*^	5(2.5~10)[36]	14(6~30)[49]	0.001^*^
Total number of doses of epinephrine	2(1~5)[85]	2(0~3)[55]	4(1~8)[30]	0.026^*^	1.5(0~2.5)[36]	3(1~8)[49]	0.010^*^
Epinephrine dosing interval, min	3.3(2.8~5)[66]	3.3(2.7~4.3)[41]	4(2.8~5.4)[25]	0.314	3.2(2.5~4.5)[26]	3.5(2.9~5.3)[40]	0.258
Number of doses of epinephrine (categorical)				0.011^*^			0.018^*^
4 or fewer	43(50.59)	32(58.18)	11(36.67)		22(61.11)	21(42.86)	
>4	23(27.06)	9(16.36)	14(46.67)		4(11.11)	19(38.78)	
None	19(22.35)	14(25.45)	5(16.67)		10(27.78)	9(18.37)	
Duration of chest compressions (categorical), min				0.018^*^			0.050
≤15	56(65.88)	42(76.36)	14(46.67)		29(80.56)	27(55.1)	
<15 ≤30	16(18.82)	8(14.55)	8(26.67)		4(11.11)	12(24.49)	
>30	13(15.29)	5(9.09)	8(26.67)		3(8.33)	10(20.41)	
Place of arrest				0.454			0.294
Emergency department	6(7.06)	5(9.09)	1(3.33)		3(8.33)	3(6.12)	
General ward	19(22.35)	11(20)	8(26.67)		5(13.89)	14(28.57)	
Intensive care unit	45(52.94)	29(52.73)	16(53.33)		19(52.78)	26(53.06)	
Operating room	8(9.41)	7(12.73)	1(3.33)		6(16.67)	2(4.08)	
OPD or other clinics	2(2.35)	1(1.82)	1(3.33)		1(2.78)	1(2.04)	
Home or non-clinical location	5(5.88)	2(3.64)	3(10)		2(5.56)	3(6.12)	
Open compression	2(2.35)	1(1.82)	1(3.33)	>0.999	1(2.78)	1(2.04)	>0.999
ECMO application	19(22.35)	10(18.18)	9(30)	0.211	4(11.11)	15(30.61)	0.033^*^
Intubation during CPR	28(32.94)	16(29.09)	12(40)	0.307	9(25)	19(38.78)	0.182
Intubation present at the time of arrest	29(34.12)	15(27.27)	14(46.67)	0.072	9(25)	20(40.82)	0.129
Etiology				0.221			0.677
Respiratory	45(52.94)	34(61.82)	11(36.67)		21(58.33)	24(48.98)	
Cardiac	16(18.82)	8(14.55)	8(26.67)		7(19.44)	9(18.37)	
Sepsis	6(7.06)	3(5.45)	3(10)		1(2.78)	5(10.2)	
Others	12(14.12)	7(12.73)	5(16.67)		4(11.11)	8(16.33)	
Unknown	6(7.06)	3(5.45)	3(10)		3(8.33)	3(6.12)	
Underlying diseases							
Pulmonary disease	27(31.76)	17(30.91)	10(33.33)	0.819	11(30.56)	16(32.65)	0.837
Neurologic disease	25(29.41)	18(32.73)	7(23.33)	0.364	11(30.56)	14(28.57)	0.843
Cardiologic disease	31(36.47)	18(32.73)	13(43.33)	0.332	14(38.89)	17(34.69)	0.691
Hemato-oncologic disease	12(14.12)	5(9.09)	7(23.33)	0.103	2(5.56)	10(20.41)	0.052
Pulmonary hypertension	9(10.59)	8(14.55)	1(3.33)	0.150	6(16.67)	3(6.12)	0.159
Others	29(34.12)	22(40)	7(23.33)	0.121	16(44.44)	13(26.53)	0.085
None	6(7.06)	5(9.09)	1(3.33)	0.417	3(8.33)	3(6.12)	0.695
SB administration during 30days after CA				0.012^*^			0.002^*^
Administered	56(65.88)	31(56.36)	25(83.33)		17(47.22)	39(79.59)	
Not administered	29(34.12)	24(43.64)	5(16.67)		19(52.78)	10(20.41)	
SB administration during CPR				0.164			0.017^*^
Administered	26(30.59)	14(25.45)	12(40.00)		6(16.67)	20(40.82)	
Not administered	59(69.41)	41(74.55)	18(60.00)		30(83.33)	29(59.18)	
SB 0 h, mEq	0(0~6)	0(0~0.2)[55]	0(0~8)	0.193	0(0~0)[36]	0(0~10)	0.019^*^
SB 24 h, mEq	21(0~160)	8(0~120)[55]	96.4(21~343)	0.001^*^	0(0~47.5)[36]	84(20~194)	0.001^*^
SB 48 h, mEq	25(0~172.9)	12(0~120)[55]	167.5(23~467.5)	<0.001^*^	0(0~111.8)[36]	120(20~363)	0.002^*^

Data are presented as number (percent) or median [IQR] as appropriate. A pediatric cerebral performance category (PCPC) score of 1 to 3 or no change in score from prearrest indicates good neurologic outcomes, whereas a PCPC score of 4 to 6 indicates poor neurologic outcomes. Day was defined as 7:00 am to 10:59 pm, and night was defined as 11:00 pm to 6:59 am. IHCA, in-hospital cardiac arrest; OHCA, out-of-hospital cardiac arrest; PEA, pulseless electrical activity; VT/VF, ventricular tachycardia/ventricular fibrillation; OPD, outpatient department; ECMO, extracorporeal membrane oxygenation; CPR, cardiopulmonary resuscitation; SB, sodium bicarbonate; CA, cardiac arrest; SB 0 h, total administered amount of sodium bicarbonate during cardiopulmonary resuscitation; SB 24 h, total administered amount of sodium bicarbonate until 24 hours after cardiac arrest; SB 48h, total administered amount of sodium bicarbonate until 48 hours after cardiac arrest. *P < 0.05.

Most of the patients had one or more underlying diseases (92.9%). Among them, 12 (14.1%) patients had underlying hemato-oncologic diseases, and all of the malignancy patients had at least more than three chemotherapy with or without hematopoietic stem cell transplantation. Their DNI values before CA (sampling within 48 hours before CA) were mostly 0 (Supplementary Table S1). About 30% of the patients had pre-existing neurological co-morbidities (29.4%) (Supplementary Table S2). Major etiology of CA was respiratory etiology (52.9%). Most cases of respiratory etiology were due to airway obstruction (68.9%, *e.g*. T-tube displacement/obstruction, laryngospasm, bronchial asthma) (Supplementary Table S3).

Modes^23^ and causes^24^ of deaths were divided into five categories (Supplementary Tables S4 and S5). The majority of deaths occurred in unstable patients with progressive, refractory hemodynamic shock, and most patients died from non-escalation of inotropics or failed resuscitation. Withdrawal of support occurred in one patient who had discontinued ECMO due to intractable multi-organ failure. Two patients who were applied ECMO received heart transplantation, and no one switched to permanent assist devices.

### DNI level and short-term mortality

**Figure 2:**
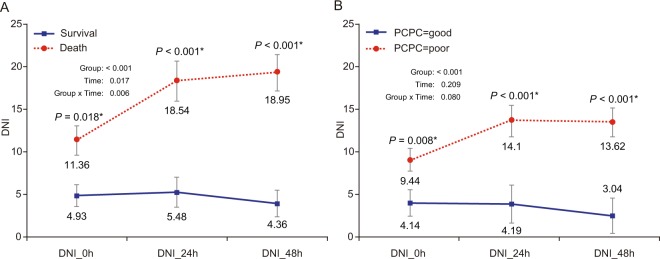
Mean DNI values over time associated with mortality (**A**) and neurologic outcomes (**B**) at 30 days after cardiac arrest. DNI, delta neutrophil index; 0 h, immediately after return of spontaneous circulation; 24 h, 24 hours after cardiac arrest; 48 h, 48 hours after cardiac arrest. PCPC, Pediatric Cerebral Performance Category. A PCPC score of 1 to 3 or no change in score from prearrest indicates good neurologic outcomes, whereas a PCPC score of 4 to 6 indicates poor neurologic outcomes (Fig. 2B).

There were significant differences in DNI values at 0 hour (immediately after ROSC), 24 hours, and 48 hours after CA and in the peak value between survival and non-survival groups in terms of 30-day mortality (Table 2). The linear-mixed model showed that DNI values were significantly different between groups over the time according to 30-day survival (group: *P* < 0.001, time: *P* = 0.017, group × time: *P* = 0.006) (Fig. 2A).

**Table 2:** Laboratory findings after cardiac arrest stratified by outcomes.

Variables	Total(N = 85) [n]	30-day mortality		*P*-value	30-day PCPC score		*P*-value
		Survival (N = 55) [n]	Death (N = 30) [n]		Good (N = 36) [n]	Poor (N = 49) [n]	
DNI 0 h, %	3.4(0~11.7)[85]	1.4(0~6.9)[55]	9.5(0~16.1)[30]	0.032^*^	1.5(0~5.6)[36]	6.1(0~13.9)[49]	0.070
DNI 24 h, %	3.6(0~14.5)[78]	1.4(0~4.7)[50]	15.1(6.8~27.7)[28]	<0.001^*^	1(0~3.3)[31]	9.4(2.4~19.3)[47]	<0.001^*^
DNI 48 h, %	4.3(0.4~13.1)[71]	3(0~5.7)[47]	12.6(4.7~28.1)[24]	<0.001^*^	1.3(0~4.3)[29]	8(2.2~21.2)[42]	<0.001^*^
DNI peak, %	16.6(5.9~32.3)[85]	10.6(4.3~17.4)[55]	37.6(19.1~51.3)[30]	<0.001^*^	7.5(3.7~13.1)[36]	21.2(16.4~47.9)[49]	<0.001^*^
WBC 0 h, 10^3^/μL	13.6(7.6~17.5)[84]	14.6(10.1~20.7)[54]	9(6.5~14.8)[30]	0.006^*^	14.8(10.4~20.5)[36]	10.6(7.3~16)[48]	0.027^*^
WBC 24 h, 10^3^/μL	11.3(6.9~16.7)[76]	10.4(7.5~16)[48]	12.1(4.7~17.2)[28]	0.944	11(7.2~16.7)[31]	11.3(5.6~16.6)[45]	0.870
WBC 48 h, 10^3^/μL	10.1(6.7~14.3)[69]	10(7.7~14.3)[45]	10.1(5.1~14.9)[24]	0.584	9.7(7.8~13.5)[29]	10.4(6.3~15.7)[40]	0.832
ANC 0 h, 10^3^/μL	9.3(6~14.8)[53]	10.8(7.6~18.4)[37]	6.1(3.8~8.5)[16]	0.005^*^	11.3(7.7~18.4)[25]	7.1(5.7~11.1)[28]	0.036^*^
ANC 24 h, 10^3^/μL	8.8(4.7~13)[50]	7.1(4.6~11.4)[36]	9.7(9.1~13.4)[14]	0.230	7.2(4.7~10.6)[23]	9.3(4.6~13.4)[27]	0.514
ANC 48, 10^3^/μL	8.8(5.4~13.2)[42]	8(4.5~12.4)[32]	10(8~18)[10]	0.136	7.6(4.5~11.3)[20]	10(6.8~18)[22]	0.155
Hb 0 h, g/dL	10.4±2.5[84]	10.5±2.6[54]	10.2±2.4[30]	0.576	10.7±2.9[36]	10.1±2.1[48]	0.313
Hb 24 h, g/dL	10.2(9~11.8)[76]	10.2(9~11.5)[48]	10.1(9.4~12.3)[28]	0.234	10.3(9.3~11.8)[31]	10.1(9~11.6)[45]	0.937
Hb 48 h, g/dL	9.8(8.9~11.2)[69]	9.6(8.7~11)[45]	10.7(9.5~11.8)[24]	0.072	9.6(8.9~11.1)[29]	9.9(9~11.2)[40]	0.563
RDW 0 h, %	15.1(13.8~17.3)[84]	15.2(13.6~17.4)[54]	14.9(13.9~17.1)[30]	0.541	15.8(13.4~18)[36]	14.8(13.9~16.4)[48]	0.509
RDW 24 h, %	15.1(14.3~17.2)[76]	15(14.3~17.2)[48]	15.2(14.3~17.1)[28]	0.867	15.8(14.1~18.3)[31]	14.8(14.3~15.9)[45]	0.203
RDW 48 h, %	15.3(14.4~16.4)[69]	15.1(14.1~16.2)[45]	15.6(14.6~17.3)[24]	0.132	15.6(14.1~17.6)[29]	15.2(14.4~16.1)[40]	0.662
Platelets 0 h, 10^3^/μL	175(111.5~337)[84]	248(158~380)[54]	124.5(48~228)[30]	0.003^*^	248(158.5~379.5)[36]	166(55.5~286.5)[48]	0.049^*^
Platelets 24 h, 10^3^/μL	126.5(83.5~248)[76]	156(110~308.5)[48]	88.5(63.5~142)[28]	<0.001^*^	155(116~316)[31]	109(71~195)[45]	0.007^*^
Platelets 48 h, 10^3^/μL	109(80~231)[69]	123(83~256)[45]	95(65~143)[24]	0.023^*^	124(94~257)[29]	101.5(70.5~149.5)[40]	0.095
CRP 0 h, mg/L	13.7(4.2~72.6)[45]	11.3(2.1~26.8)[29]	49.9(6.6~122.5)[16]	0.043^*^	9.3(1.4~24)[16]	21.6(6.1~78)[29]	0.115
CRP 24 h, mg/L	40.6(4.4~77.9)[23]	40.6(4.4~77.1)[13]	38(5.2~139.2)[10]	0.598	43.7(4.4~77.9)[9]	35.7(5.2~73.7)[14]	0.975
CRP 48 h, mg/L	68.8±62.1[17]	73.7±70.2[12]	57.1±40.5[5]	0.631	91.1±87.2[7]	53.2±33.6[10]	0.309
PT 0 h, sec	15.6(12.8~21.9)[78]	15.3(12.4~19.6)[52]	17.1(14.4~23)[26]	0.103	19.3±18.4[33]	21.6±22.1[45]	0.001^*^
PT 24 h, sec	16.4(13.9~22)[58]	15.9(13.7~20.7)[37]	19.3(15.2~22.8)[21]	0.097	16.2±4.8[24]	20.6±7.7[34]	0.046^*^
PT 48 h, sec	17(13~21.9)[54]	15.1(12.5~19.7)[36]	21.4(18~25.2)[18]	0.007^*^	14.3(12.5~16.3)[22]	19.1(15.6~23.8)[32]	0.032^*^
INR 0 h, INR	1.4(1.1~1.9)[78]	1.3(1.1~1.7)[52]	1.5(1.3~2)[26]	0.100	1.3(1.1~1.8)[33]	1.4(1.3~2)[45]	0.079
INR 24 h, INR	1.5(1.2~1.9)[58]	1.4(1.2~1.8)[37]	1.7(1.4~2.1)[21]	0.096	1.3(1.2~1.5)[24]	1.7(1.4~2.1)[34]	0.008^*^
INR 48 h, INR	1.5(1.2~2)[54]	1.4(1.1~1.8)[36]	1.9(1.6~2.2)[18]	0.005^*^	1.3(1.1~1.4)[22]	1.7(1.4~2.1)[32]	0.022^*^
aPTT 0 h, sec	49(34.7~78.6)[75]	45.3(33~77.1)[51]	63.1(36.7~82.7)[24]	0.214	38.2(32~66.7)[32]	53.3(39.4~81.6)[43]	0.057
aPTT 24 h, sec	37.4(31.7~50.5)[58]	37.4(31.8~50.6)[37]	37.4(31.7~46.8)[21]	0.808	36.6(30.9~54.6)[24]	38.1(33.2~46.8)[34]	0.764
aPTT 48 h, sec	38.8(32.3~47.7)[53]	37.6(32~47.7)[35]	41.3(37~54.6)[18]	0.182	37.1(32.1~47.7)[21]	39.7(35.2~49.8)[32]	0.561
pH 0 h	7.2±0.2[74]	7.2±0.2[46]	7.1±0.2[28]	0.069	7.3(7.1~7.4)[29]	7.1(7~7.2)[45]	0.001^*^
pH 24 h	7.4±0.1[63]	7.4±0.1[39]	7.4±0.1[24]	0.167	7.4(7.4~7.5)[25]	7.4(7.3~7.4)[38]	0.065
pH 48 h	7.4(7.3~7.5)[59]	7.4(7.4~7.5)[38]	7.4(7.3~7.4)[21]	0.154	7.4(7.4~7.5)[24]	7.4(7.3~7.5)[35]	0.046^*^
Lactate 0 h, mmol/L	10.6±5.6[54]	9.9±5.8[36]	12±4.9[18]	0.201	8.8±6[21]	11.8±5[33]	0.059
Lactate 24 h, mmol/L	2.2(1.3~7.5)[26]	1.6(1.1~2)[15]	8.2(4.1~12.8)[11]	0.001^*^	1.2(1.1~1.6)[9]	5.7(2~11.3)[17]	0.002^*^
Lactate 48 h, mmol/L	1.3(0.9~2.8)[19]	1(0.9~1.3)[12]	3(2.4~7.1)[7]	<0.001^*^	1(0.8~1.3)[7]	2.3(1.1~3.1)[12]	0.020^*^
BUN 0 h, mg/dL	13.9(9.1~24.6)[81]	12.3(8.2~21.1)[51]	17.4(10.5~26.2)[30]	0.116	12.7(8.6~23.7)[32]	14.7(9.7~24.6)[49]	0.632
BUN 24 h, mg/dL	17.8(11.2~26.3)[61]	15.2(9.1~26.3)[37]	19.6(14.5~26.6)[24]	0.065	14(8.3~23.6)[24]	19(12.4~26.9)[37]	0.073
BUN 48 h, mg/dL	14.9(9.1~22.1)[53]	14.4(8.8~22.1)[35]	15.8(12.2~22.7)[18]	0.560	14.9(7.1~25.5)[19]	14.6(9.8~22.1)[34]	0.933
Cr 0 h, mg/dL	0.6(0.3~0.8)[81]	0.5(0.3~0.7)[51]	0.7(0.4~0.9)[30]	0.027^*^	0.5(0.3~0.7)[32]	0.6(0.4~0.8)[49]	0.392
Cr 24 h, mg/dL	0.7(0.4~0.9)[61]	0.6(0.2~0.8)[37]	0.8(0.7~1.2)[24]	0.019^*^	0.6(0.2~0.8)[24]	0.7(0.5~1)[37]	0.192
Cr 48 h, mg/dL	0.6(0.2~1.1)[53]	0.6(0.2~0.8)[35]	0.6(0.3~1.4)[18]	0.336	0.6(0.3~1.1)[19]	0.5(0.2~1.1)[34]	0.428
Albumin 0 h, g/dL	3(2.3~3.3)[76]	3.1(2.5~3.6)[47]	2.6(2.2~3)[29]	0.009^*^	3.1±0.7[29]	2.7±0.7[47]	0.043^*^
Albumin 24 h, g/dL	3.2±0.5[58]	3.2±0.5[35]	3.2±0.6[23]	0.817	3.2±0.5[23]	3.2±0.6[35]	0.856
Albumin 48 h, g/dL	3.2±0.5[54]	3.2±0.5[36]	3.3±0.6[18]	0.388	3.1±0.5[21]	3.3±0.6[33]	0.246
Glucose 0 h, mg/dL	204(108~296)[75]	210(137~296)[49]	186.5(98~284)[26]	0.473	207(144~296)[30]	191(102~289)[45]	0.559
Glucose 24 h, mg/dL	124(100~160)[69]	128(97~149)[43]	122(104~161)[26]	0.985	131(97~145)[25]	122(102.5~162.5)[44]	0.847
Glucose 48 h, mg/dL	127(111~156)[59]	127(116~170)[38]	127(101~138)[21]	0.087	129(120.5~195.5)[20]	127(104~148)[39]	0.124
Sodium 0 h, mmol/L	140.8±6.9[77]	139.1±6.6[51]	144±6.5[26]	0.003^*^	139(137~141)[32]	142(139~147)[45]	0.004^*^
Sodium 24 h, mmol/L	141(139~145)[72]	140.5(137.5~144)[48]	142(141~150)[24]	0.018^*^	140(137~143)[29]	142(140~148)[43]	0.008^*^
Sodium 48 h, mmol/L	140(137~143)[64]	139(137~143)[43]	141(138~149)[21]	0.107	139.5±4.5[26]	141.7±7.6[38]	0.145

Data are presented as median [IQR] or mean ± standard deviation as appropriate. A pediatric cerebral performance category (PCPC) score of 1 to 3 or no change in score from prearrest indicates good neurologic outcomes, whereas a PCPC score of 4 to 6 indicates poor neurologic outcomes. DNI, delta neutrophil index; 0 h, immediately after return of spontaneous circulation; 24 h, 24 hours after cardiac arrest; 48h, 48hours after cardiac arrest; Peak DNI, highest DNI value during 30 days after cardiac arrest; WBC, white blood cell count; ANC, absolute neutrophil count; Hb, haemoglobin; RDW, red cell distribution width; CRP, C-reactive protein; PT, prothrombin time; INR, the international normalized ratio; aPTT, activated partial thromboplastin time; BUN, blood urea nitrogen; Cr, creatinine. *P <0.05.

DNI values at 0 hour, 24 hours, and 48 hours and peak DNI value were related to increased risk for death within 30 days after CA on univariate Cox regression analyses (Table 3). Along with DNI values, longer time of CPR; higher doses of epinephrine (especially more than four doses of epinephrine given during CPR); SB administration within 30 days after CA; intubation at the time of arrest; lower white blood cell count, absolute neutrophil count, platelet count, and albumin level; and higher lactate, prothrombin time (PT), international normalized ratio (INR), sodium, and C-reactive protein level were significantly associated with short-term mortality in univariate Cox analysis (Table 3).

**Table 3:** Cox proportional hazard analysis for 30-day mortality.

Variables	HR	(95% CI)	*P*-value
**Univariate Cox analysis**			
Compression time, min	1.02	(1.01-1.03)	<0.001^*^
Total number of doses of adrenaline	1.06	(1.02-1.1)	0.002^*^
Adrenaline number (categorical, two)			
None	Reference		
4 or fewer	0.97	(0.34-2.79)	0.951
>4	3.00	(1.08-8.33)	0.035^*^
Duration of chest compressions (categorical), min			
≤15	Reference		
<15 ≤ 30	2.38	(1.00-5.69)	0.050^*^
>30	3.29	(1.38-7.85)	0.007^*^
Intubation present at the time of arrest			
No	Reference		
Yes	2.05	(1.00-4.22)	0.050^*^
SB administration within 30 days after cardiac arrest			
Not administered	Reference		
Administered	3.20	(1.22-8.36)	0.018^*^
WBC 0 h (10^3^/μL)	0.94	(0.89-0.99)	0.014^*^
ANC 0 h (10^3^/μL)	0.90	(0.82-0.98)	0.018^*^
Platelet counts 0 h (10^3^/μL)	0.99	(0.99-1.00)	0.011^*^
Platelet counts 24 h (10^3^/μL)	0.99	(0.99-1.00)	0.002^*^
CRP 0 h (mg/L)	1.01	(1.00-1.01)	0.012^*^
PT 48h (sec)	1.06	(1.01-1.12)	0.021^*^
INR 48h (INR)	2.21	(1.2-4.09)	0.012^*^
Lactate 24 h (mmol/L)	1.45	(1.21-1.75)	<0.001^*^
Lactate 48h (mmol/L)	1.46	(1.13-1.9)	0.005^*^
Albumin 0 h (g/dL)	0.57	(0.36-0.88)	0.012^*^
Sodium 0 h (mmol/L)	1.10	(1.04-1.16)	0.001^*^
Sodium 24 h (mmol/L)	1.09	(1.03-1.16)	0.005^*^
Sodium 48h (mmol/L)	1.08	(1.01-1.15)	0.028^*^
DNI 0 h (%)	1.06	(1.03-1.09)	<0.001^*^
DNI 24 h (%)	1.04	(1.02-1.06)	<0.001^*^
DNI 48h (%)	1.07	(1.04-1.09)	<0.001^*^
DNI peak (%)	1.05	(1.03-1.07)	<0.001^*^
DNI 0 h (%)			
<8.8	Reference		
≥8.8	3.16	(1.53-6.53)	0.002^*^
DNI 24 h (%)			
<3.3	Reference		
≥3.3	9.72	(2.93-32.3)	<0.001^*^
DNI 48h (%)			
<6.5	Reference		
≥6.5	5.13	(2.12-12.43)	<0.001^*^
DNI peak (%)			
<19.1	Reference		
≥19.1	6.30	(2.69-14.75)	<0.001^*^
**Multivariable Cox analysis**			
CPR duration	1.02	(1.01-1.04)	0.003^*^
Sodium 0 h	1.07	(1.01-1.14)	0.024^*^
DNI 24 h			
<3.3	Reference		
≥3.3	11.80	(2.73-51.01)	<0.001^*^
**C-index**			**0.799**

HR, hazard ratio; CI, confidence interval; SB, sodium bicarbonate; WBC, white blood cell; ANC, absolute neutrophil count; CRP, C-reactive protein; PT, prothrombin time; INR, international normalized ratio; DNI, delta neutrophil index; 0 h, immediately after return of spontaneous circulation; 24 h, 24 hours after cardiac arrest; 48h, 48 hours after cardiac arrest; Peak DNI, highest DNI value during 30 days after cardiac arrest; CPR, cardiopulmonary resuscitation *P <0.05.

The optimal DNI cut-off values to predict 30-day mortality were 8.8% at 0 hour, 3.3% at 24 hours, 6.5% at 48 hours, and 19.1% of the peak value. DNI ≥ 3.3% at 24hours showing high sensitivity (89.3%) and moderate specificity (66.0%) predicted 30-day mortality after pediatric CA (Table 4). Multivariable analysis showed that if DNI at 24 hours was ≥3.3%, then the risk for 30-day mortality increased by 11-fold [hazard ratio (HR): 11.8, 95% confidence interval (CI): 2.73–51.01; *P* < 0.001]. It also indicated that 30-day mortality increased by 7% or 2% for a one-unit increase in sodium at 0 hour and the duration of CPR, respectively (HR: 1.07, 95% CI: 1.01–1.14; *P* = 0.024 and HR: 1.02, 95% CI: 1.01–1.04; *P* = 0.003) (Table 3).

**Table 4:** Sensitivity and specificity of DNI for the prediction of outcome according to cut-off values. A pediatric cerebral performance category (PCPC) score of 1 to 3 or no change in score from prearrest indicates good neurologic outcomes, whereas a PCPC score of 4 to 6 indicates poor neurologic outcomes. DNI, delta neutrophil index; 0 h, immediately after return of spontaneous circulation; 24 h, 24 hours after cardiac arrest; 48h, 48 hours after cardiac arrest; Peak DNI, highest DNI value during 30 days after cardiac arrest.

DNI (cut off value)	Sensitivity (95% CI)	Specificity (95% CI)
**Outcome (30-day mortality)**		
DNI 0 h (≥8.8%)	56.7(37.4–74.5)	78.2(65.0––88.2)
DNI 24 h (≥3.3%)	89.3(71.8–97.7)	66.0(51.2–78.8)
DNI 48h (≥6.5%)	70.8(48.9–87.4)	78.7(64.3–89.3)
DNI peak (≥19.1%)	76.7(57.7–90.1)	78.2(57.7–90.1)
**Outcome (30-day poor neurologic outcome)**		
DNI 0 h (>8.1%)	46.9(32.5–61.7)	83.3(67.2–93.6)
DNI 24 h (>3.3%)	70.2(55.1–82.7)	77.4(58.9–90.4)
DNI 48h (>4.3%)	69.1(52.9–82.4)	82.8(64.2–94.2)
DNI peak (>14.5%)	75.5(61.1–86.7)	80.6(64.0–91.8)

**Figure 3:**
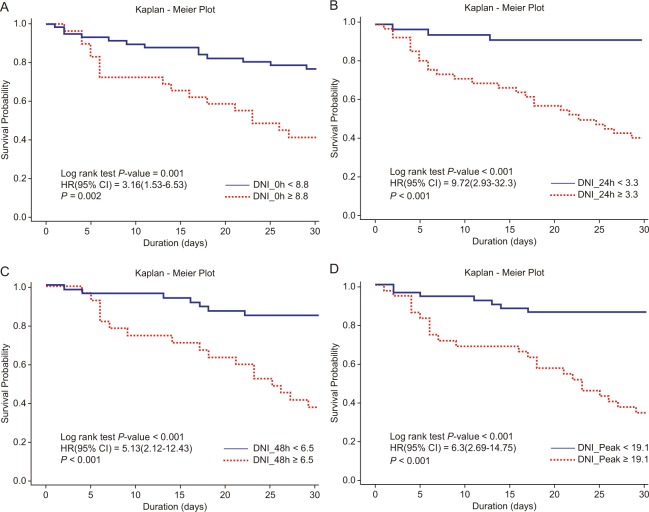
DNI as a predictor of 30-day mortality after cardiac arrest. Higher DNI values at immediately after return of spontaneous circulation (**A**), 24 hours (**B**), and 48 hours (**C**) after cardiac arrest and the peak value (highest DNI value over 30 days after cardiac arrest) were significantly associated with predicted increases in 30-day mortality risk among pediatric patients with cardiac arrest.

Study participants were divided into two groups based on the cut-off values. Kaplan–Meier curves revealed the survival curve in each group (Fig. 3). Patients with DNI values higher than the respective cut-off values at 0 hour, 24 hours, and 48 hours and the peak value had significantly shorter survival lengths in log-rank test (at 0 hour: *P* = 0.002; at 24 hours, 48 hours and peak value: *P* < 0.001).

### DNI level and survival to discharge

We performed the analysis based on survival to discharge (Supplementary Table S6). Among the 85 patients initially included in the current study, the number of subjects who survived to discharge was 46 (54.1%). Three patients suffered ≥2 CA during the study period (at least 30 days apart), and we counted their death or discharge as one event; as a result, three cases were excluded due to duplication. Finally, 82 cases were included for further analysis.

Lower DNI values at 24 hours and 48 hours and peak DNI value were associated with survival to discharge after CA on univariate Cox regression analyses. If DNI at 48 hours was ≥6.5%, then the risk for survival to discharge decreased by 0.37-fold [hazard ratio (HR): 0.37, 95% confidence interval (CI): 0.16-0.9; *P* = 0.027] (Supplementary Table S6).

### DNI level and short-term neurologic outcome

The mean DNI values at 24 hours, 48 hours and the peak DNl value were also significantly higher with poor neurologic outcomes (defined as PCPC score = 4–6) (Table 2). In addition, there were significant differences in DNI value between the good and poor neurologic outcomes groups, but not according to time (group: *P* < 0.001, time: *P* = 0.209, group × time: *P* = 0.080) (Fig. 2B).

Univariate logistic regression analyses showed higher DNI values at 0 hour, 24 hours, and 48 hours and for peak value were significantly associated with increased risks of poor neurologic outcomes (Table 5). Lower pH, platelet count, and albumin level, as well as higher PT, INR, and sodium level were also associated with poor neurologic outcomes in univariate logistic analysis (Table 5). In addition, the administration of SB during CPR and within 30 days after CA were significantly associated with increased risks of poor neurologic outcomes in univariate logistic analysis, respectively (Table 5).

**Table 5:** Logistic regression analysis for 30-day neurologic outcome.

Variables	OR	(95% CI)	*P*-value
**Univariate logistic analysis**			
Compression time, min	1.03	(1.00-1.06)	0.037^*^
Adrenaline number (categorical, two)			
None	Reference		
4 or fewer	1.06	(0.36-3.13)	0.915
>4	5.28	(1.30-21.5)	0.020^*^
ECMO application			
No	Reference		
Yes	3.53	(1.06-11.76)	0.040^*^
SB administration within 30 days after cardiac arrest			
Not administered	Reference		
Administered	4.36	(1.68-11.32)	0.003^*^
SB administration during CPR			
Not administered	Reference		
Administered	3.45	(1.21-9.81)	0.020^*^
Platelet counts 24 h (10^3^/μL)	0.99	(0.99-1.00)	0.022^*^
PT 24 h (sec)	1.14	(1.02-1.28)	0.026^*^
INR 24 h (INR)	5.17	(1.31-20.47)	0.019^*^
INR 48h (INR)	3.94	(1.13-13.81)	0.032^*^
pH 0 h	0.01	(<0.01-0.19)	0.002^*^
pH 24 h	0.00	(<0.01-1.07)	0.053
pH 48h	<0.01	(<0.01-0.23)	0.019^*^
Albumin 0 h, g/dL	0.49	(0.25-0.99)	0.048^*^
Sodium 0 h, mmol/L	1.09	(1.01-1.18)	0.028^*^
Sodium 24 h, mmol/L	1.14	(1.03-1.27)	0.011^*^
DNI 0 h (%)	1.08	(1.01-1.15)	0.022^*^
DNI 24 h (%)	1.09	(1.02-1.15)	0.009^*^
DNI 48h (%)	1.16	(1.05-1.29)	0.004^*^
DNI peak (%)	1.09	(1.04-1.14)	<0.001^*^
DNI 0 h (%)			
≤8.1	Reference		
>8.1	4.42	(1.56-12.52)	0.005^*^
DNI 24 h (%)			
≤3.3	Reference		
>3.3	8.08	(2.83-23.06)	<0.001^*^
DNI 48h (%)			
≤4.3	Reference		
>4.3	10.71	(3.34-34.3)	<0.001^*^
DNI peak (%)			
≤14.5	Reference		
>14.5	12.77	(4.46-36.54)	<0.001^*^
**Multivariable logistic analysis**			
PH 0 h	0.01	(<0.01-0.22)	0.006^*^
Sodium 0 h	1.06	(0.96-1.16)	0.233
DNI 24 h			
≤3.3	Reference		
>3.3	8.04	(2.06-31.42)	0.003^*^
**AUC**			**0.871**

OR, odds ratio; CI, confidence interval; AUC, area under the curve; ECMO, extracorporeal membrane oxygenation; SB, sodium bicarbonate; CPR, cardiopulmonary resuscitation; PT, prothrombin time; INR, the international normalized ratio; DNI, delta neutrophil index; 0 h, immediately after return of spontaneous circulation; 24 h, 24 hours after cardiac arrest; 48h, 48 hours after cardiac arrest; Peak DNI, highest DNI value during 30 days after cardiac arrest. *P < 0.05.

The optimal DNI cut-off values to predict poor neurologic outcomes were 8.1% at 0 hour, 3.3% at 24 hours, 4.3% at 48 hours, and 14.5% of the peak value in our study. DNI > 3.3% at 24 hours showing moderate sensitivity (70.2%) and moderate specificity (77.4%) predicted poor neurologic outcome after pediatric CA (Table 4). Multivariable logistic regression using these cut-off values indicated that a DNI value of >3.3% at 24 hours holding pH and sodium at 0 hour was associated with an eight-fold (OR: 8.04; 95% CI: 2.06–31.42; *P* = 0.003) OR for poor neurologic outcomes. In addition, as pH at 0 hour decreased, the OR of poor neurologic outcome increased significantly (OR: 0.01; 95% CI: < 0.01–0.22; *P* = 0.006) (Table 5).

We performed further analysis for neurologic outcomes among the survivors after CA. CPR duration, total number of doses of epinephrine, and total amount of SB administration during CPR were significantly higher in poor neurologic outcome patients (Supplementary Table S7). Mean pH level at 0 hour and median Cr level at 48 hours were significantly lower in poor neurologic outcome group, and median sodium level at 0 hour was higher in poor neurologic outcome group (Supplementary Table S8).

Lower level of Ph at 0 hour and the administration of SB during CPR were significantly associated with poor neurologic outcomes in univariate analysis (Supplementary Table S9). In addition, DNI value >4.3% at 48 hours and peak DNI value >14.5% during 30 days after CA were also significantly associated with poor neurologic outcomes [odds ratio (OR), 4.60 and 4.67, respectively]. However, we could not find such correlation in multivariable analysis (Supplementary Table S9).

## Discussion

In the present study, we demonstrated that DNI could be a significant independent predictor of 30-day mortality and neurologic outcomes in children in the early post-resuscitation period. Concretely, a DNI value of higher than 3.3% at 24 hours after CA significantly predicted 30-day mortality (HR: 11.8; *P* < 0.001) and neurologic outcomes (OR: 8.04; *P* = 0.003) in this study group of pediatric patients.

In our study, DNI value was significantly higher in non-survivors than in survivors from the time immediately after CA to later. After CA, patients experienced “post-cardiac arrest syndrome,” which mimics the physiologic changes consistent with observations of severe sepsis^1,25^. Ischemia-reperfusion after CA stimulates innate immunity, and subsequent cytokine release mediates sterile systemic inflammatory responses and multi-organ dysfunction^16,25^. Previously, Yune *et al*. reported that DNI values of more than 8.4% immediately after emergency department (ED) admission (HR: 3.22) and those of more than 12.9% at 24 hours after ED admission (HR: 3.29) were associated with 30-day mortality in adult OHCA patients^20^.

### DNI and ischemia-reperfusion injury after cardiac arrest

The DNI indicates the ratio of circulating immature granulocytes to total neutrophil count. DNI is calculated by using an automated blood cell analyzer^26^ with two separate channels, myeloperoxidase (MPO) channels and nuclear lobularity channels^17^. By subtracting the fraction of mature polymorphonuclear leukocytes from the sum of MPO-reactive cells, it can estimate the fraction of immature granulocytes such as metamyelocytes, myeloctyes, and promyelocytes^17^.

High DNI indicate an increase in circulating immature granulocytes, and it occurs in various acute conditions such as acute hematological malignancies, bleeding, and sepsis^18^. Previous studies have also described DNI as a useful marker for diagnosing sepsis and septic shock, predicting positive blood culture and disseminated intravascular coagulation^17,18,28^. Similar to DNI, the elevated immature/total granulocyte ratio (IG%), referred to as a left-shift^27^, is another index of increasing immature granulocytes. Sauneuf *et al*. reported that a higher immature/total granulocyte ratio at ICU admission independently predicted death or poor neurologic outcomes (CPC 3–5) in a large prospective cohort of patients after OHCA^29^.

Post-cardiac arrest syndrome is a second, complex phase after CA^1,25^. Systemic ischemia/reperfusion is one of the key components of this status^30^. Systemic ischemia/reperfusion induces a whole-body inflammation similar to SIRS^1,31^. Increase in circulating immature granulocytes in early post-CA period has been reported^20,29^, and it is assumed to be due to various mechanisms, such as bone marrow ischemia^32^, neutrophil paralysis^33^, endotoxemia^34^, and a compensatory response due to depletion of mature neutrophils in bone marrow^35^.

In this study, we used an automated blood cell analyzer (ADVIA 2120) to determine DNI, which overcomes the limitation of manual counting that is strongly correlated with manual immature granulocyte counts^17,20^. Yune *et al*. used the same ADVIA 2120 analyzer, and demonstrated that DNI were associated with 30-day mortality in adult OHCA (cut-off value of 12.9% at 24 hours after ED admission)^20^.

While most published studies used ADVIA to measure DNI for assessing the proportion of immature granulocytes^36^, the majority of them focused on sepsis or infection-related diseases. Since we could only find one study that evaluated the clinical value of DNI as diagnostic or prognostic marker after CA^20^, more studies on how DNI proportionately reflects the ischemic insult after CA should be performed using large cohorts.

In contrast with DNI, our results showed that white blood cell count (WBC) and absolute neutrophil count (ANC) at 0 hour were lower in patients who died compared to survivors. A previous study also reported that initial blood tests during resuscitation revealed a significantly lower WBC in the sustained ROSC group of adult OHCA patients^37^. In another retrospective single-center study with adult IHCA and OHCA patients who underwent targeted temperature management for post cardiac arrest syndrome within 6 hours of CA, WBC and median ANC were also significantly lower in decedents than in survivors^38^.

Ischemia/reperfusion after CA activates immunological process, resulting in elevated levels of cytokines, soluble receptors, and endotoxins; and these changes are associated with clinically poor outcomes^1,25^. In sepsis, the overproduction of nitric oxide (NO), chemokines, and cytokines reduces chemotaxis and adhesion interactions between neutrophils and the endothelium. Alves-Filho *et al*. suggest neutrophil paralysis to be characterized by the failure of neutrophils to migrate into the site of infection, and inappropriate neutrophil sequestration in remote organs^39^. In post-cardiac arrest syndrome, similar to SIRS^1^, elevated P- and E-selectin, soluble vascular cell adhesion molecule-1, and soluble intercellular molecule-1 were observed after CA^25,33^. Although the mechanisms underlying the decrease in WBC and ANC in contrast to immature granulocytes during early resuscitation period are unclear, neutrophil paralysis in post-cardiac arrest syndrome can be a possible explanation; severe ischemia may induce more degree of interactions between adhesion molecules and activated neutrophils with subsequently more decreased circulating neutrophils^20^.

In summary, whole-body ischemia/reperfusion after CA results in a status similar to sepsis-like syndrome. It also elevates cytokines, the presence of endotoxin in plasma, activation of coagulation pathways, and inhibition of anticoagulant pathways^1,30,40^. However, pediatric studies evaluating immune-inflammatory response that represents systemic ischemia/reperfusion status after CA are limited in comparison to adult studies^41^. Therefore, data extrapolated from adult studies are currently used for the understanding and management of pediatric post-cardiac arrest syndrome^30,42^.

In our study, patients who had higher values than the cut-off for DNI at each time point showed significantly shorter survival times in Kaplan–Meier curves. Sol *et al*. reported that DNI at the time of pediatric intensive care unit (ICU) admission was associated with disease severity and mortality, with the cut-off value to predict mortality being 4.95%^43^. The cut-off value of DNI at 24 hours for predicting 30-day mortality in our study was similar to the results from the aforementioned pediatric ICU patients.

Nevertheless, the cut-off value at 24 hours in our research was much lower than that of adult OHCA patients (3.3% vs. 12.9%). This discrepancy may be attributed to the differences among enrolled patients. Our subjects were mostly pediatric in-hospital CA (IHCA) patients. Compared to OHCA, IHCA patients tend to have a lower risk of ischemic damage due to the early recognition of CA and administration of high-quality CPR^44,45^. Consequently, the lower cut-off value of DNI in our study may reflect the lower degree of ischemic insult. However, there has been no research about the relationship between the degree of ischemia and DNI in pediatric patients. Therefore, further research is required demonstrate how inflammatory response is reflected by DNI and how it differs and between adults and children in post-cardiac arrest status.

Until now, DNI has been primarily known as a useful diagnostic and prognostic biomarker in bacteraemia and sepsis patients. Park *et al*. reported that a DNI value of more than 6.5% was a useful laboratory marker for differentiating severe sepsis and septic shock in adult ICU patients^18^. Hence, the DNI values in patients whose CA aetiology was presumed to be sepsis might be high before arrest, and also affect the association between DNI level and outcomes. Nevertheless, in our study, the mean DNI value was not different between sepsis-associated and other aetiology groups at each time point (Supplementary Table S10).

Furthermore, other infectious diseases caused respiratory failure (e.g. pneumonia) may likely influence circulating leukocytes; however, DNI usually rises in more severe infections, such as sepsis or severe systemic inflammatory status^18,46^. For example, previous studies have shown that DNI could be useful for distinguishing between various infectious conditions, such as APN from lower UTI (cut-off value, >1.3%)^47^, pulmonary tuberculosis from community acquired pneumonia (CAP) (cut-off value, ≤1.0%)^48^, and low-grade CAP from upper respiratory infection (cut-off value, >1.7%)^49^. However, their cut-off values were much lower than for those for sepsis (cut-off value, > 6.5%)^18^, bacteremia (cut-off value, 4.4%)^46^ or prognostic cut-off value of adult OHCA (cut-off value, >8.4%)^20^ with our result (cut-off value, 3.3%).

Furthermore, CRP at 0 hour can be raised due to a severe infection, but it also can be raised in the days following resuscitation of CA^50,51^, reflecting a systemic inflammatory response. Previous studies have shown that PCT and CRP increase after adult CA, but neither PCT nor CRP level was associated with infection^52–55^.

In the present study, most cases of respiratory etiology were due to airway obstruction (68.9%, e.g. T-tube displacement/obstruction, laryngospasm, bronchial asthma). Therefore, the higher CRP at 0 hour in patients who died within 30 days may not be due to infectious cause, but rather due to their severe systemic inflammatory response.

### Underlying diseases

Another factor that might influence the circulating immature granulocytes was underlying hematologic malignancies. In our study, most patients with hematologic malignancies had at least more than three chemotherapy and/or hematopoietic stem cell transplantation. Furthermore, DNI value before CA (sampling within 48 hours before CA) were mostly 0. Therefore, we assumed that DNI could be used to evaluate of the severity of ischemic insult by reflecting the systemic inflammatory status arising from ischemic-reperfusion during post-cardiac arrest status. Unfortunately, the number of samples that were taken within 48 hours before CA was so small that we could not perform sub-group analysis.

### Other prognostic factors

Along with DNI value, a longer duration of CPR and higher sodium at 0 hour also showed a significant association with mortality in our multivariable logistic regression model. Longer duration of compression has been shown to be associated with decreased survival in both pediatric IHCA and OHCA patients^4,8^. In our study, CPR duration was also significantly shorter in survivors. Furthermore, CPR lasting more than 30 minutes was significantly associated with 30-day mortality in univariate analysis.

Makino *et al*. found that 105 adult OHCA patients who were admitted to the ED had no difference in sodium level compared to control patients^56^. Separately, Shin *et al*.^57^ reported that, among the initial blood laboratory parameters present during CPR of adult OHCA patients, sodium level showed no significant difference between survival and non-survival groups. In comparison, higher sodium level was associated with mortality after pediatric CA in our study.

In fact, the sodium level might have a relationship to the SB administered. SB administration has been considered a treatment option for severe metabolic acidosis in CA. The use of SB during a CA is also known to be associated with increased mortality^8^, but it remains controversial with regard to pediatric patients^58^. In our study, the sodium level was significantly higher at 0 hour and 24 hours in the death group. However, the total administered amount of SB was not significantly different at 0 hour, but was significantly higher at 24 hours and 48 hours in the death group. Only the administration of SB within 30 days after CA was significantly associated with the 30-days mortality in the univariate cox analysis.

So far, to the best of our knowledge, there has been no specific study that investigated the association between sodium level and prognosis after CA. Therefore, future studies are needed to specify and confirm the relationship between serum sodium level and outcomes after CA.

### DNI value and neurologic outcomes

In our study, DNI was significantly higher in cases of poor neurologic outcome at each time point. The association between DNI and neurologic outcome in various conditions, including early post-resuscitation status, has rarely been studied, although one single-center retrospective adult OHCA study showed that DNI values of more than 8.4% at ED admission (HR: 2.718; *P* < 0.001) and those of more than 10.5% at 24 hours after ED admission (HR: 1.709; *P* = 0.02) were associated with poor neurologic outcomes for OHCA survivors^20^.

The cut-off level of DNI was almost similar to that in our study in the very early stage (8.4% vs. 8.1%) after CA; however, it was still higher than our result at 24 hours after CA (10.5% vs. 3.3%). As mentioned above, these findings may be due to not only the difference between enrolled patients (OHCA vs. IHCA), but also due to the neuronal vulnerability of children to hypoxic-ischemic insult^59^. Children have increased cerebral blood flow and higher metabolic needs as compared to adults, and they undergo neuronal maturation and synaptogenesis at the time of insult^42^. Therefore, lower DNI, which may reflect lower degree of inflammatory status (ischemia) in the early ROSC status, may lead to poorer neurologic outcomes in children. However, this phenomenon needs to be studied further.

There have been discrepant results regarding the correlation between pH and neurologic outcomes in CA. In a recent cohort study of pediatric IHCA patients, those with poor neurologic outcomes showed lower pH levels according to the PCPC scale^14^. Similarly, in our study, lower pH at 0 hour was independently associated with short-term poor neurologic outcomes. However, the other report involving pediatric OHCA patients showed that pH level was not related to neurological recovery based on the Vineland Adaptive Behavior Scale, Second Edition (VABS-II) score^60^. The research protocols, such as cohort design, outcome assessment methods, and place of CA, could contribute to or account for these differences.

DNI can be determined with a CBC without adding costs or time. Moreover, DNI could be performed independently and irrespectively of clinical conditions. Therefore, DNI could be a quick and inexpensive method to estimate prognosis in children after CA, especially in the context of limited resources. However, a combination of other prognostic parameters might increase outcome prediction, so clinicians should consider multiple factors when predicting outcomes in infants and children after CA^2,6,14,60,61^.

## Limiations

Our study had several limitations. First, this was a retrospective study performed at a single, tertiary hospital. Further multicenter studies are required to validate our results. Second, we were not able to assess long-term clinical outcomes. Third, we could not compare the superiority between DNI and other known prognostic biomarkers, such as NSE (neuron-specific enolase**)** and SB100. Further prospective studies are needed to identify the accuracy and usefulness of these prognostic markers in pediatric CA patients. Fourth, we could not evaluate the pre-existence of infectious disease, which could affect the DNI value during early post-arrest status. However, there were no differences between sepsis-associated aetiology group and the other aetiology group at each time point. Therefore, we can assume that sepsis status prior to CA has little effect on DNI values during the early post-resuscitation period. We could not analyze the previous level of DNI or CRP level before CA; therefore, even if DNI is drawn immediately after CA, it might be influenced by previous infectious condition.

Although DNI can contribute to survival prognostication or neuroprognostication in pediatric CA, decision-making for an individual patient by DNI alone can be inappropriate, as it only provides the probability for a dichotomous outcome (*e.g*. death or survival). In addition, DNI has yet to be validated in prospective pediatric studies after CA. Currently, no single variable has been found to be sufficiently accurate and reliable for prognostication in children after CA. Practitioners should consider multiple factors when predicting outcomes in infants and children who achieve ROSC after CA^2,30^.

In summary, we found that a DNI value during early stage of CA independently predicts not only 30-days mortality and survival to discharge, but also neurologic outcome at 30 days after CA in pediatric patients. Since DNI can be measured concomitantly with CBC, it can be a rapid and easy method with high sensitivity and moderate specificity for predicting mortality after pediatric CA. It may be especially useful in clinical setting with limited source. We suggest that pediatric patients with higher DNI values during the early post-resuscitation period be carefully monitored, so that proper management could be carried out when necessary, especially if the DNI value is higher than 3.3% at 24 hours after CA.

## Methods

### Study design, population, and setting

This retrospective observational cohort study was performed between January 2012 and January 2018 at a single tertiary referral hospital. We reviewed medical records from our hospital’s CA registry, investigating all pediatric patients who had achieved ROSC after CPR. We included patients who had survived for at least 24 hours after ROSC and received CPR for 2 minutes or more. If patients suffered more than one event of CA during 30 days, we only included the first event. If CA occurred at least 30 days apart, we considered it a separate event. Exclusion criteria were as follows: age older than 19 years, lack of laboratory data (less than two serum DNI values), and loss to follow-up. The management of patients with CA and post-resuscitation care was performed according to the 2010 and 2015 European Resuscitation Council/American Heart Association guidelines^2,62^.

The main study outcomes were 30-day mortality and PCPC score at 30 days after CA^63^. A PCPC score of 1 to 3 or no change in score from the time of pre-arrest indicated good neurologic outcomes, whereas a PCPC score of 4 to 6 indicated poor neurologic outcomes^12,64^. Additionally, we assigned a PCPC score of 6 to patients who died within 30 days after CA^20^. Follow-up of patients who were discharged within 30 days was conducted by a review of subsequent out-patient-department clinic charts or through phone interviews with caregivers. All PCPC scores were assigned by chart review. Two reviewers, (SHY) and (JGA), independently scored; and in cases of disagreement, a third reviewer (JAL) was consulted. Assigning investigators were blinded to DNI results before scoring.

This study was approved by the Institutional Review Board of Yonsei University Health System (4-2018-0632), and the requirement for consent from the patients was waived.

### Data collection

The patient data collected were as follows: demographics, underlying significant comorbidities, aetiology of arrest, presence of endotracheal tube at the time of arrest, intubation during the compression, initial arrest rhythm, duration of chest compressions, doses of epinephrine during CPR, epinephrine dosing interval (duration of CPR/doses of epinephrine during CPR), administration of SB during CPR and within 30 days after CA, total amount of sodium bicarbonate administered during CPR, 24 and 48 hours after CA, use of open-chest cardiac compressions, application of extracorporeal membrane oxygenation at the time of ROSC, time of arrest (day was defined as 7:00 am to 10:59 pm and night was defined as 11:00 pm to 6:59 am), and location of arrest^7,8,61^.

Following ROSC, routine blood sampling was performed in all patients according to our resuscitation protocols. DNI and other laboratory tests were measured immediately after ROSC (0 hour) as well as at 24 hours and 48 hours after CA. Peak DNI (the highest DNI value over 30 days after CA) was then determined. In most cases, samples were collected at a fixed time according to the protocol. However, if sampling was difficult for certain patients, several laboratory tests could not go on time along with DNI. Selection of laboratory data was based on the presence of DNI test in time.

CBC, including DNI value, was analyzed using an automated blood cell analyzer (ADVIA 2120; Siemens, Munich, Germany). DNI value was determined by the following formula: DNI = (neutrophil subfraction + eosinophil subfraction measured in the myeloperoxidase channel) − (polymorphonuclear subfraction measured in the nuclear lobularity channel)^17,18^.

### Statistical analysis

Continuous variables are presented in the format of mean ± standard deviation or median (Q1–Q3), depending on whether the normality assumption was met or not. If the normality assumption was not violated, then an independent two-sample t-test was used to compare outcomes between groups. In the case of violation of the normality assumption, comparison was conducted using Mann–Whitney U test. Categorical variables were compared by chi-squared test or Fisher’s exact test, as appropriate, and the results were reported as a frequency along with the percentage in parenthesis.

Cox proportional hazard regression analysis was conducted to investigate the effects of variables on predicting 30-day mortality. Multiple Cox proportional hazard regression analysis was performed using a stepwise method for variable selection to identify independent prognostic factors. Harrell’s C index value also was listed for the assessment of prediction performance, with higher C values indicating good performance on prediction. Multicollinearity was checked based on the variable inflation factor (VIF) of each variable included in the model. Variables with a VIF of ≥10 were not included in the same model due to multicollinearity.

Contal and O’Quigley’s technique was used to find the optimal cut-off point for time to events data. The optimal cut-off point was determined by maximizing the test statistics of log-rank test. Kaplan–Meier curves were constructed according to 30-day mortality.

Logistic regression analysis was performed to identify the significant factors for neurologic outcomes. Multivariable logistic regression analysis was performed using a stepwise method for variable selection to determine if DNI level was independently associated with poor outcomes. Receiver operating characteristic (ROC) analyses were performed to examine the performances of DNI levels in predicting poor neurologic outcomes. Statistical analyses were performed using SAS version 9.4 (SAS Institute Inc., Cary, NC, USA) and MedCalc Statistical Software version 18.9.1 (MedCalc Software, Ostend, Belgium). *P* < 0.05 was considered to be statistically significant.

## Supplementary information

Supplementary information.

## Data Availability

The datasets used and/or analyzed in the current study are available from the corresponding author upon reasonable request.
