# Supplementary material for: Prognostic value of the delta neutrophil index in pediatric cardiac arrest
Source: Sci Rep. 2020 Feb 26;10:3497. doi: 10.1038/s41598-020-60126-y (PMC7044231; doi:10.1038/s41598-020-60126-y)
Supplement: Supplementary file 1 — Supplementary information. [file 41598_2020_60126_MOESM1_ESM.docx]

**Supplementary Information**

**Prognostic value of the delta neutrophil index in pediatric cardiac arrest**

*Seo Hee Yoon, MD^1^; Eun Ju Lee, MS^2^; Jinae Lee, PhD^2^; Moon Kyu Kim, MD^1^ and Jong Gyun Ahn, MD^1^*

1. Department of Pediatrics, Severance Children’s Hospital, Yonsei University College of Medicine, Seoul, Korea
2. Department of Biomedical Systems Informatics, Biostatistics Collaboration Unit, Yonsei University College of Medicine, Seoul, Korea

**Corresponding Author: *Jong Gyun Ahn, MD****
Department of Pediatrics, Severance Children’s Hospital, Yonsei University College of Medicine, 50-1 Yonsei-ro, Seodaemun-gu, Seoul 03722, Korea
Email: JGAHN@yuhs.ac
Tel: +82-2-2228-2057
Fax: +82-2-393-9118

**Supplementary Table S1. Underlying hemato-oncologic disease and delta neutrophil index values**

| **No.** | **Underlying hemato-oncologic disease** | **DNI_previous  (within 48hrs before CA)** | **DNI_0h** |
| --- | --- | --- | --- |
| 1 | Choroidal plexus tumor | 19.5 | 16.1 |
| 2 | Langerhans cell histiocytosis | 0.0 | 0 |
| 3 | Burkitt Lymphoma | none | 47.3 |
| 4 | Hodgkin's lymphoma | none | 23.2 |
| 5 | Hemophilia B | 0.0 | 8.8 |
| 6 | Atypical teratoid/ rhabdoid tumor, brain | none | 13.9 |
| 7 | ALL (testicular relapse, Rt. Scrotum) | 0.0 | 0 |
| 8 | Sarcoma, neck | none | 1.4 |
| 9 | Rhabdomyosarcoma (retroperitoneal) | none | 6.1 |
| 10 | T-lymphoblastic leukemia | 4.5 | 8.1 |
| 11 | Non-Hodgkins lymphoma | 0.0 | 0.7 |
| 12 | Neuroblastoma | none | 1.2 |

DNI, delta neutrophil index

**Supplementary Table S2. Pre-existing neurological co-morbidities and neurologic outcome at 30 days after cardiac arrest**

| **Pre-existing neurological co-morbidities (n=25)** | **N(%)** |
| --- | --- |
| Cerebral palsy | 3(12%) |
| Cerebrovascular accident | 1(4%) |
| Encephalitis | 3(12%) |
| Encephalopathy | 3(12%) |
| Epilepsy | 5(20%) |
| Hydranencephaly | 1(4%) |
| Metachromatic leukodystrophy | 1(4%) |
| Mitochondrial cytopathy | 3(12%) |
| Myasthenia gravis | 1(4%) |
| Myotonic disorder | 1(4%) |
| Myotubular myopathy | 1(4%) |
| Spinal muscular atrophy | 2(8%) |
| **Neurologic outcome among the pre-existing neurological co-morbidities** |  |
| Good | 11(44%) |
| Poor | 14(56%) |
| **PCPC score among the pre-existing neurological co-morbidities (average)** |  |
| Pre-arrest | 3.84 |
| Post-arrest (30 days after cardiac arrest) | 4.80 |
| ΔPCPC | 0.96 |

PCPC, Pediatric Cerebral Performance Category; ΔPCPC, change in the PCPC. A PCPC score of 1 to 3 or no change in score from prearrest indicates good neurologic outcomes, whereas a PCPC score of 4 to 6 worsen than previous PCPC score indicates poor neurologic outcomes.

**Supplementary Table S3. Respiratory causes leading to cardiac arrest (n=45)**

| **Respiratory causes leading to cardiac arrest (n=45)** | **N (%)** |
| --- | --- |
| Decreased respiratory effort (e.g. overdose of narcotic and sedative) | 6(13.3) |
| Upper airway obstruction (e.g. laryngospasm, T-tube displacement/obstruction, asphyxia) | 23(51.1) |
| Lower airway obstruction (e.g. bronchial asthma) | 8(17.8) |
| Abnormities of the alveoli (aspiration, pulmonary hemorrhage, severe pneumonia) | 8(17.8) |
| - Acute aspiration | 3(6.7) |
| - Pulmonary hemorrhage | 1(2.2) |
| - Severe pneumonia | 4(8.9) |
| Total | 45(100) |

**Supplementary Table S4. Modes of deaths**

| **Modes of deaths** | **N (%)** |
| --- | --- |
| Withdrawal of life-sustaining technologies (withdrawal) | 1(2.8) |
| Non-escalation | 17(47.2) |
| Failed resuscitation | 13(36.1) |
| Code-then-withdrawal | 0(0.0) |
| Death by neurologic criteria | 5(13.9) |
| **Total** | **36**^†^ **(100)** |

^†^Among the 85 cases initially included in the current study, three cases suffered ≥2 CA during the study period (at least 30 days apart), and we counted their death or discharge as one event; as a result, three cases were excluded due to duplication. Finally, the number of subjects who survived to discharge was 46 and did not survive to discharge was 36, respectively.

**Supplementary Table S5. Categorization of reasons for death following cardiac arrest**

| **Categorization of reasons for death following cardiac arrest** | **N (%)** |
| --- | --- |
| Progressive, refractory hemodynamic shock | 16(44.4) |
| Respiratory failure | 4(11.1) |
| Sudden cardiac death | 9(25.0) |
| Neurological withdrawal of care | 5(13.9) |
| Comorbid withdrawal of care | 2(5.6) |
| **Total** | **36**^†^ **(100)** |

^†^Among the 85 cases initially included in the current study, three cases suffered ≥2 CA during the study period (at least 30 days apart), and we counted their death or discharge as one event; as a result, three cases were excluded due to duplication. Finally, the number of subjects who survived to discharge was 46 and did not survive to discharge was 36, respectively.

**Supplementary Table S6. Cox regression analysis for survival to discharge**

| **Variables** | **HR** | **(95% CI)** | ***P*-value** |
| --- | --- | --- | --- |
| **Univariate Cox analysis** |  |  |  |
| Compression time, min | 0.990 | (0.97-1.00) | 0.124 |
| Total number of doses of epinephrine | 0.970 | (0.92-1.02) | 0.236 |
| Epinephrine number (categorical, two) |  |  |  |
| None | Reference |  |  |
| 4 or fewer | 0.590 | (0.28-1.23) | 0.157 |
| >4 | 0.490 | (0.20-1.21) | 0.124 |
| Duration of chest compressions (categorical), min |  |  |  |
| ≤15 | Reference |  |  |
| <15 ≤30 | 0.560 | (0.24-1.28) | 0.167 |
| >30 | 0.640 | (0.24-1.66) | 0.354 |
| Intubation present at the time of arrest |  |  |  |
| No | Reference |  |  |
| Yes | 0.800 | (0.42-1.53) | 0.494 |
| SB administration during 30days after cardiac arrest |  |  |  |
| Not administered | Reference |  |  |
| Administered | 0.430 | (0.24-0.80) | **0.007^*^** |
| SB administration during CPR |  |  |  |
| Not administered | Reference |  |  |
| Administered | 0.830 | (0.44-1.56) | 0.557 |
| WBC 0h, 10^3/μL | 0.989 | (0.96-1.01) | 0.395 |
| ANC 0h, 10^3/μL | 1.035 | (0.99-1.08) | 0.144 |
| Platelets 0h 10^3/μL | 1.001 | (1.00-1.00) | 0.548 |
| Platelets 24h, 10^3/μL | 1.002 | (1.00-1.004) | 0.132 |
| CRP 0h, mg/L | 0.997 | (0.991-1.003) | 0.281 |
| PT 48h, sec | 0.916 | (0.84-0.995) | **0.038^*^** |
| INR 48h, INR | 0.345 | (0.13-0.92) | **0.034^*^** |
| Lactate 24h, mmol/L | 0.914 | (0.65-1.28) | 0.601 |
| Lactate 48h, mmol/L | 0.819 | (0.22-3.05) | 0.766 |
| Albumin 0h, g/dL | 1.154 | (0.74-1.81) | 0.535 |
| Sodium 0h, mmol/L | 0.951 | (0.91-0.999) | **0.045^*^** |
| Sodium 24h, mmol/L | 0.93 | (0.87-0.99) | **0.022^*^** |
| Sodium 48h, mmol/L | 0.983 | (0.92-1.05) | 0.574 |
| DNI 0h, % | 0.956 | (0.91-1.01) | 0.075 |
| DNI 24h, % | 0.953 | (0.91-0.995) | **0.030^*^** |
| DNI 48h, % | 0.906 | (0.85-0.97) | **0.005^*^** |
| DNI peak, % | 0.952 | (0.92-0.98) | **0.001^*^** |
| SB 0h, mEq | 0.999 | (0.99-1.01) | 0.702 |
| SB 24h, mEq | 0.999 | (0.998-1.001) | 0.471 |
| SB 48h, mEq | 0.999 | (0.998-1.001) | 0.421 |
| DNI 0h, % |  |  |  |
| <8.8 | Reference |  |  |
| ≥8.8 | 0.510 | (0.23-1.14) | 0.100 |
| DNI 24h, % |  |  |  |
| <3.3 | Reference |  |  |
| ≥3.3 | 0.520 | (0.27-1.02) | 0.059 |
| DNI 48h, % |  |  |  |
| <6.5 | Reference |  |  |
| ≥6.5 | 0.370 | (0.16-0.90) | **0.027^*^** |
| DNI peak, % |  |  |  |
| <19.1 | Reference |  |  |
| ≥19.1 | 0.350 | (0.16-0.75) | **0.007^*^** |
| **Multivariable Cox analysis** |  |  |  |
| CPR duration | 0.990 | (0.97-1.01) | 0.213 |
| Sodium 0h | 0.980 | (0.92-1.04) | 0.461 |
| DNI 24h |  |  |  |
| <3.3 | Reference |  |  |
| ≥3.3 | 0.520 | (0.24-1.10) | 0.085 |
| **C-index** | 0.679 | | |

HR, hazard ratio; CI, confidence interval; WBC, white blood cell; ANC, absolute neutrophil count; CPR, cardiopulmonary resuscitation; DNI, delta neutrophil index; 0h, immediately after return of spontaneous circulation; 24h, 24 hours after cardiac arrest; 48h, 48 hours after cardiac arrest; Peak DNI, highest DNI value during 30 days after cardiac arrest; SB, sodium bicarbonate; SB 0h, total administered amount of sodium bicarbonate during cardiopulmonary resuscitation; SB 24h, total administered amount of sodium bicarbonate until 24hrs after cardiac arrest; SB 48h, total administered amount of sodium bicarbonate until 48hrs after cardiac arrest. **P* < 0.05.

**Supplementary Table S7. Baseline clinical characteristics among the survivors stratified by neurologic outcomes**

| **Variables** | **Total( N=48**^†^**)** **[n]** | **30-day PCPC score** | | ***p*-value** |
| --- | --- | --- | --- | --- |
|  |  | **Good (N=35)** **[n]** | **Poor (N=13)** **[n]** |  |
| Time of arrest |  |  |  | 0.458 |
| Day | 37(77.08) | 28(80) | 9(69.23) |  |
| Night | 11(22.92) | 7(20) | 4(30.77) |  |
| Female sex (n,%) | 14(29.17) | 11(31.43) | 3(23.08) | 0.728 |
| Age, years | 4.96(1.13~8.65)[48] | 3.62(1.07~10.15)[35] | 2.01(1.84~3.59)[13] | 0.441 |
| IHCA/OHCA |  |  |  | >0.999 |
| In Hospital | 46(95.83) | 33(94.29) | 13(100) |  |
| Out of Hospital | 2(4.17) | 2(5.71) | - |  |
| Initial rhythm (n,%) |  |  |  | 0.780 |
| Asystole | 11(22.92) | 9(25.71) | 2(15.38) |  |
| PEA | 13(27.08) | 10(28.57) | 3(23.08) |  |
| VT/VF | 5(10.42) | 4(11.43) | 1(7.69) |  |
| Bradycardia | 17(35.42) | 11(31.43) | 6(46.15) |  |
| Unknown | 2(4.17) | 1(2.86) | 1(7.69) |  |
| CPR duration, min | 14.94(3~17)[48] | 5(2~10)[35] | 14(8~28)[13] | **0.016^*^** |
| Total number of doses of epinephrine | 3.81(0.5~4)[48] | 1(0~2)[35] | 4(1~9)[13] | **0.034^*^** |
| Epinephrine dosing interval, min | 4.2(2.58~4.25)[36] | 3.1(2.5~4.5)[25] | 3.13(3~4)[11] | 0.597 |
| Number of doses of epinephrine (categorical) | |  |  | 0.145 |
| 4 or fewer | 27(56.25) | 21(60) | 6(46.15) |  |
| >4 | 9(18.75) | 4(11.43) | 5(38.46) |  |
| none | 12(25) | 10(28.57) | 2(15.38) |  |
| Duration of chest compressions (categorical), min | |  |  | 0.151 |
| ≤15 | 35(72.92) | 28(80) | 7(53.85) |  |
| <15 ≤30 | 8(16.67) | 4(11.43) | 4(30.77) |  |
| >30 | 5(10.42) | 3(8.57) | 2(15.38) |  |
| Place of arrest |  |  |  | 0.852 |
| Emergency department | 5(10.42) | 3(8.57) | 2(15.38) |  |
| General ward | 8(16.67) | 5(14.29) | 3(23.08) |  |
| Intensive care unit | 25(52.08) | 18(51.43) | 7(53.85) |  |
| Operating room | 7(14.58) | 6(17.14) | 1(7.69) |  |
| OPD or Other clinics | 1(2.08) | 1(2.86) | - |  |
| Home or Non-clinical location | 2(4.17) | 2(5.71) | - |  |
| Open compression | 1(2.08) | 1(2.86) | - | >0.999 |
| ECMO apply | 8(16.67) | 4(11.43) | 4(30.77) | 0.187 |
| Intubation during CPR | 15(31.25) | 9(25.71) | 6(46.15) | 0.293 |
| Intubation present at the time of arrest | 13(27.08) | 8(22.86) | 5(38.46) | 0.298 |
| Etiology |  |  |  | 0.363 |
| Respiratory | 28(58.33) | 20(57.14) | 8(61.54) |  |
| Cardiac | 8(16.67) | 7(20) | 1(7.69) |  |
| Sepsis | 3(6.25) | 1(2.86) | 2(15.38) |  |
| Others | 6(12.5) | 4(11.43) | 2(15.38) |  |
| Unknown | 3(6.25) | 3(8.57) | - |  |
| SB administration during CPR |  |  |  | **0.005^*^** |
| Administered | 14(29.17) | 6(17.14) | 8(61.54) |  |
| Not administered | 34(70.83) | 29(82.86) | 5(38.46) |  |
| SB 0h, mEq | 15.61(0~5.8)[48] | 0(0~0)[35] | 10(0~21.4)[13] | **0.006^*^** |
| SB 24h, mEq | 96.7(0~120)[48] | 0(0~55)[35] | 84(0~120)[13] | 0.108 |
| SB 48h, mEq | 121.22(0~121.75)[48] | 0(0~123.5)[35] | 96(0~120)[13] | 0.207 |

Data are presented as number (percent) or median [IQR] as appropriate. A pediatric cerebral performance category (PCPC) score of 1 to 3 or no change in score from prearrest indicates good neurologic outcomes, whereas a PCPC score of 4 to 6 indicates poor neurologic outcomes. Day was defined as 7:00 am to 10:59 pm and night was defined as 11:00 pm to 6:59 am. IHCA, in-hospital cardiac arrest; OHCA, out-of-hospital cardiac arrest; CPR, cardiopulmonary resuscitation; PEA, pulseless electrical activity; VT/VF, ventricular tachycardia/ ventricular fibrillation; OPD, outpatient department; ECMO, extracorporeal membrane oxygenation; SB, sodium bicarbonate; SB 0h, total administered amount of sodium bicarbonate during cardiopulmonary resuscitation; SB 24h, total administered amount of sodium bicarbonate until 24hrs after cardiac arrest; SB 48h, total administered amount of sodium bicarbonate until 48hrs after cardiac arrest. ^†^Among the 85 cases initially included in the current study, 48 cases survived to discharge. If cardiac arrest occurred at least 30 days apart, we considered it a separate event. **P* < 0.05.

**Supplementary Table S8. Laboratory findings among the survivors stratified by neurologic outcomes**

| **Variables** | **Total(N=48^†^)[n]** | **30-day PCPC score** | | ***p*-value** |
| --- | --- | --- | --- | --- |
|  |  | **Good (N=35)[n]** | **Poor (N=13)[n]** |  |
| DNI 0h, % | 4.25(0~5.9)[48] | 1.3(0~5.7)[35] | 0(0~6.1)[13] | 0.511 |
| DNI 24h, % | 4.22(0~4)[43] | 1.1(0~3.3)[30] | 1.1(0~4)[13] | 0.713 |
| DNI 48h, % | 3.75(0~5.3)[40] | 2.15(0~4.3)[28] | 3.65(0~9.4)[12] | 0.503 |
| DNI peak, % | 11.91(3.85~17.2)[48] | 8.1(3.6~13.6)[35] | 16.4(9.3~17.8)[13] | 0.093 |
| WBC 0h, 10^3/μL | 16.6(9.82~19.98)[48] | 14.64(10.11~20.22)[35] | 13.76(7.44~16.4)[13] | 0.620 |
| WBC 24h, 10^3/μL | 12.71(7.4~15.49)[42] | 10.52(7.19~16.57)[30] | 10.38(7.46~14.87)[12] | 0.803 |
| WBC 48h, 10^3/μL | 11.26(7.18~14.3)[39] | 9.57(7.26~13.5)[28] | 9.21(7.18~15.09)[11] | 0.938 |
| ANC 0h, 10^3/μL | 12.22(6.64~16.74)[33] | 11.18(7.16~17.88)[24] | 8.73(6.22~10.82)[9] | 0.292 |
| ANC 24h, 10^3/μL | 9.26(4.64~10.46)[32] | 6.91(4.7~10.31)[22] | 6.86(4.57~12.1)[10] | 0.904 |
| ANC 48, 10^3/μL | 8.92(4~11.5)[27] | 7.46(4.14~11.5)[19] | 7.13(2.09~14.89)[8] | 0.773 |
| Hb 0h, g/dL | 10.59±2.69[48] | 10.75±2.94[35] | 10.18±1.87[13] | 0.526 |
| Hb 24h, g/dL | 10.23(9~11.5)[42] | 10.35(9.3~11.8)[30] | 9.2(8.85~10.65)[12] | 0.189 |
| Hb 48h, g/dL | 9.94(8.7~11)[39] | 9.6(8.85~11.2)[28] | 9.6(8.6~9.8)[11] | 0.387 |
| RDW 0h, % | 15.82(13.65~17.5)[48] | 15.8(13.3~18.1)[35] | 14.8(14.2~15.1)[13] | 0.229 |
| RDW 24h, % | 15.82(14.2~17.2)[42] | 15.85(14.1~18.3)[30] | 14.7(14.3~14.95)[12] | 0.099 |
| RDW 48h, % | 15.64(13.9~17.1)[39] | 15.6(14.1~17.6)[28] | 14.7(13.8~15.1)[11] | 0.083 |
| Platelets 0h, 10^3/μL | 259.94±148.47[48] | 255.66±144.68[35] | 271.46±163.8[13] | 0.747 |
| Platelets 24h, 10^3/μL | 218.19(111~285)[42] | 153(116~285)[30] | 153.5(105~305)[12] | 0.912 |
| Platelets 48h, 10^3/μL | 191.54(94~256)[39] | 123.5(94~250)[28] | 148(81~256)[11] | 0.745 |
| ESR 0h, mm/hr | 16.17(2~20.5)[24] | 3(2~17)[15] | 2(2~24)[9] | 0.875 |
| ESR 24h, mm/hr | 23.71(2~20)[7] | 16(2~20)[5] | 6.5(3~10)[2] | 0.852 |
| ESR 48h, mm/hr | 3.25(2~4.5)[4] | 4(4~4)[1] | 2(2~5)[3] | >0.999 |
| CRP 0h, mg/L | 34.92(2.2~35.6)[26] | 9.3(1.4~23.95)[16] | 23.28(6.7~47.9)[10] | 0.208 |
| CRP 24h, mg/L | 47.84±49.41[11] | 53.65±52.48[9] | 21.7±26.72[2] | 0.437 |
| CRP 48h, mg/L | 73±77.51[10] | 91.13±87.18[7] | 30.7±19.86[3] | 0.283 |
| PT 0h, sec | 19.11(12.4~19.5)[45] | 15.1(12.15~20.5)[32] | 15.1(13~18.6)[13] | 0.700 |
| PT 24h, sec | 17.14(13.45~20.65)[32] | 15(13.3~17)[23] | 16.3(13.7~21.8)[9] | 0.312 |
| PT 48h, sec | 16.29(12.5~19)[30] | 14.4(12.5~16.3)[21] | 15.9(13~19)[9] | 0.737 |
| INR 0h, INR | 1.69(1.09~1.7)[45] | 1.32(1.07~1.81)[32] | 1.31(1.16~1.65)[13] | 0.691 |
| INR 24h, INR | 1.51(1.17~1.82)[32] | 1.29(1.16~1.48)[23] | 1.43(1.26~1.92)[9] | 0.275 |
| INR 48h, INR | 1.43(1.16~1.67)[30] | 1.27(1.16~1.42)[21] | 1.4(1.16~1.67)[9] | 0.703 |
| aPTT 0h, sec | 57.4(33~68)[44] | 37.3(31.8~74.9)[31] | 49(40.5~61.1)[13] | 0.205 |
| aPTT 24h, sec | 45.36(31.3~50.7)[32] | 37.1(30.4~54.8)[23] | 38.8(33.7~41.1)[9] | 0.771 |
| aPTT 48h, sec | 44.74(32.1~47.5)[29] | 37.95(32.2~47.85)[20] | 37.6(32.1~40.7)[9] | 0.779 |
| D-dimer 0h, ng/mL | 3913.08(787~5712)[25] | 1750(1063~3492)[13] | 4233.5(449.5~7219)[12] | 0.648 |
| D-dimer 24h,ng/mL | 10829.57(515~2650)[7] | 769(515~2650)[6] | 1687(1687~1687)[1] | 0.811 |
| D-dimer 48h, ng/mL | 6390.83(374~3404)[6] | 706(374~2738)[5] | 3404(3404~3404)[1] | 0.584 |
| pH 0h | 7.23±0.19[39] | 7.27±0.17[28] | 7.11±0.21[11] | **0.017^*^** |
| pH 24h | 7.42±0.09[33] | 7.43±0.08[24] | 7.39±0.13[9] | 0.374 |
| pH 48h | 7.41(7.38~7.47)[31] | 7.42(7.38~7.47)[23] | 7.42(7.37~7.46)[8] | 0.671 |
| Lactate 0h, mmol/L | 9.32±5.68[29] | 8.65±6.13[20] | 10.8±4.48[9] | 0.355 |
| Lactate 24h, mmol/L | 2.15(1.1~1.9)[13] | 1.2(1.1~1.55)[8] | 1.9(1.7~2)[5] | 0.293 |
| Lactate 48h, mmol/L | 1.14±0.35[10] | 1.07±0.28[6] | 1.25±0.45[4] | 0.446 |
| BUN 0h, mg/dL | 21.03(8.65~22.95)[44] | 12.7(9.1~25.3)[31] | 10.8(8.2~20.8)[13] | 0.394 |
| BUN 24h, mg/dL | 16.76(8.2~23.6)[32] | 15.2(9.4~26.3)[23] | 12.1(7.3~17.8)[9] | 0.648 |
| BUN 48h, mg/dL | 15.76(9~21.1)[29] | 16.45(8.8~25.5)[18] | 9.8(9~19.4)[11] | 0.464 |
| Cr 0h, mg/dL | 0.89(0.26~0.71)[44] | 0.49(0.28~0.71)[31] | 0.45(0.25~0.61)[13] | 0.482 |
| Cr 24h, mg/dL | 0.69(0.2~0.75)[32] | 0.58(0.2~0.84)[23] | 0.46(0.2~0.66)[9] | 0.403 |
| Cr 48h, mg/dL | 0.66(0.24~0.69)[29] | 0.64(0.3~1.06)[18] | 0.24(0.2~0.62)[11] | **0.040^*^** |
| Albumin 0h, g/dL | 3.04±0.65[40] | 3.06±0.68[28] | 2.97±0.59[12] | 0.668 |
| Albumin 24h, g/dL | 3.2±0.5[30] | 3.16±0.47[22] | 3.3±0.58[8] | 0.503 |
| Albumin 48h, g/dL | 3.19±0.46[30] | 3.12±0.47[20] | 3.33±0.42[10] | 0.232 |
| Glucose 0h, mg/dL | 242.98(137~309)[42] | 204(144~296)[29] | 243(136~309)[13] | 0.746 |
| Glucose 24h, mg/dL | 144.75(100.5~156.5)[36] | 129.5(95~145)[24] | 146(107~169)[12] | 0.235 |
| Glucose 48h, mg/dL | 153.23(113~170)[31] | 131(123~212)[19] | 124(109~158.5)[12] | 0.191 |
| Sodium 0h, mmol/L | 139.8(137~142.5)[44] | 139(137~141)[31] | 144(141~147)[13] | **0.014^*^** |
| Sodium 24h, mmol/L | 141.1±5.47[41] | 140.11±5.37[28] | 143.23±5.25[13] | 0.089 |
| Sodium 48h, mmol/L | 139.03(137~141)[36] | 139(137~141)[25] | 138(137~140)[11] | 0.421 |

Data are presented as median [IQR] or mean ± standard deviation as appropriate. A pediatric cerebral performance category (PCPC) score of 1 to 3 or no change in score from prearrest indicates good neurologic outcomes, whereas a PCPC score of 4 to 6 indicates poor neurologic outcomes. DNI, delta neutrophil index; 0h, immediately after return of spontaneous circulation; 24h, 24 hours after cardiac arrest; 48h, 48hours after cardiac arrest; Peak DNI, highest DNI value during 30 days after cardiac arrest; WBC, white blood cell count; ANC, absolute neutrophil count; Hb, haemoglobin; RDW, red cell distribution width; ESR, erythrocyte sedimentation rate; CRP, C-reactive protein; PT, prothrombin time; INR, the international normalized ratio; aPTT, activated partial thromboplastin time; BUN, blood urea nitrogen; Cr, creatinine. ^†^Among the 85 cases initially included in the current study, 48 cases survived to discharge. If cardiac arrest occurred at least 30 days apart, we considered it a separate event. **P* < 0.05.

**Supplementary Table S9. Logistic regression analysis for 30-day neurologic outcomes after cardiac arrest (survivors only)**

| **Variables** | **OR** | **(95% CI)** | ***p*-value** |
| --- | --- | --- | --- |
| **Univariate logistic analysis** |  |  |  |
| Compression time, min | 1.030 | (1.00-1.00) | 0.081 |
| Epinephrine number (categorical, two) |  |  |  |
| None | Reference |  |  |
| 4 or fewer | 1.430 | (0.24-8.38) | 0.693 |
| >4 | 6.250 | (0.84-46.57) | 0.074 |
| ECMO apply |  |  |  |
| No | Reference |  |  |
| Yes | 3.440 | (0.72-16.00) | 0.123 |
| Platelets 24h, 10^3/μL | 1.000 | (1.00-1.01) | 0.866 |
| PT 24h, sec | 1.090 | (0.96-1.24) | 0.201 |
| INR 24h, INR | 3.050 | (0.63-14.65) | 0.164 |
| INR 48h, INR | 0.990 | (0.15-6.60) | 0.992 |
| pH 0h | 0.010 | (<0.001-0.59) | **0.028^*^** |
| pH 24h | 0.020 | (<0.001-83.97) | 0.366 |
| pH 48h | 0.000 | (<0.001-49.13) | 0.242 |
| Albumin 0h, g/dL | 0.790 | (0.28-2.00) | 0.659 |
| Sodium 0h, mmol/L | 1.120 | (0.99-1.26) | 0.073 |
| Sodium 24h, mmol/L | 1.120 | (0.98-1.28) | 0.104 |
| DNI 0h, % | 1.010 | (0.92-1.11) | 0.831 |
| DNI 24h, % | 1.000 | (0.92-1.08) | 0.907 |
| DNI 48h, % | 1.090 | (0.95-1.25) | 0.221 |
| DNI peak, % | 1.040 | (0.98-1.10) | 0.205 |
| SB administration during CPR |  |  |  |
| Not administered | Reference |  |  |
| Administered | 7.733 | (1.866-32.047) | **0.005^*^** |
| SB 0h, mEq | 1.003 | (0.991-1.014) | 0.647 |
| SB 24h, mEq | 1.001 | (0.998-1.004) | 0.610 |
| SB 48h, mEq | 1.000 | (0.998-1.003) | 0.815 |
| DNI 0h, % |  |  |  |
| ≤8.1 | Reference |  |  |
| >8.1 | 0.880 | (0.15-5.02) | 0.885 |
| DNI 24h, % |  |  |  |
| ≤3.3 | Reference |  |  |
| >3.3 | 2.054 | (0.506-8.341) | 0.314 |
| DNI 48h, % |  |  |  |
| ≤4.3 | Reference |  |  |
| >4.3 | 4.600 | (1.04-20.38) | **0.045^*^** |
| DNI peak, % |  |  |  |
| ≤14.5 | Reference |  |  |
| >14.5 | 4.670 | (1.19-18.35) | **0.028^*^** |
| **Multivariable logistic analysis** |  |  |  |
| pH 0h | 0.015 | (<0.001, 2.593) | 0.110 |
| Sodium 0h | 1.128 | (0.967, 1.316) | 0.124 |
| DNI 24h |  |  |  |
| ≤3.3 | Reference |  |  |
| >3.3 | 1.942 | (0.301, 12.515) | 0.485 |
| **AUC** |  |  | 0.826 |
|  |  |  |  |
| **Multivariable logistic analysis** |  |  |  |
| pH 0h | 0.018 | (<0.001, 4.041) | 0.146 |
| Sodium 0h | 1.117 | (0.929, 1.344) | 0.240 |
| DNI 48h, % |  |  |  |
| ≤4.3 | Reference |  |  |
| >4.3 | 1.579 | (0.190,13.112) | 0.672 |
| **AUC** |  |  | 0.831 |
|  |  |  |  |
| **Multivariable logistic analysis** |  |  |  |
| pH 0h | 0.024 | (<0.001, 3.891) | 0.150 |
| Sodium 0h | 1.147 | (0.976, 1.347) | 0.096 |
| DNI peak, % |  |  |  |
| ≤14.5 | Reference |  |  |
| >14.5 | 1.299 | (0.198, 8.510) | 0.785 |
| **AUC** |  |  | 0.855 |

OR, odds ratio; CI, confidence interval; AUC, area under the curve; CPR, cardiopulmonary resuscitation; ECMO, extracorporeal membrane oxygenation; PT, prothrombin time; INR, the international normalized ratio; DNI, delta neutrophil index; 0h, immediately after return of spontaneous circulation; 24h, 24 hours after cardiac arrest; 48h, 48 hours after cardiac arrest; Peak DNI, highest DNI value during 30 days after cardiac arrest; SB, sodium bicarbonate; SB 0h, total administered amount of sodium bicarbonate during cardiopulmonary resuscitation; SB 24h, total administered amount of sodium bicarbonate until 24hrs after cardiac arrest; SB 48h, total administered amount of sodium bicarbonate until 48hrs after cardiac arrest. **P* < 0.05.

**Supplementary Table S10. Delta neutrophil index in patients in the sepsis-associated and other aetiology of cardiac arrest groups**

| **Variables** | **Total (N=85)** | **Etiology** | | ***P*-value** |
| --- | --- | --- | --- | --- |
|  |  | **Sepsis (N=6)** | **Others (N=79)** |  |
| DNI 0h, % | 3.4(0-11.7) | 13.5(6.9-21.3) | 2.5(0-10.45) | 0.066 |
| DNI 24h, % | 3.6(0-14.5) | 13.65(3.3-37.3) | 3.4(0-13.9) | 0.108 |
| DNI 48h, % | 4.3(0.4-13.1) | 13.3(5.0- 34.7) | 4.1(0.4-11.0) | 0.156 |
| DNI peak, % | 16.6(5.9-32.3) | 25.2(9.3-60.3) | 16.6(5.6-31.6) | 0.247 |

Continuous variables are reported as median with interquartile range. DNI, delta neutrophil index; 0h, immediately after return of spontaneous circulation; 24h, 24 hours after cardiac arrest; 48h, 48 hours after cardiac arrest; Peak DNI, highest DNI value during 30 days after cardiac arrest.
